# Nutrition of Preterm Infants and Raw Breast Milk-Acquired Cytomegalovirus Infection: French National Audit of Clinical Practices and Diagnostic Approach

**DOI:** 10.3390/nu10081119

**Published:** 2018-08-18

**Authors:** Anne-Aurelie Lopes, Valerie Champion, Delphine Mitanchez

**Affiliations:** 1 Pediatric Emergency Department, AP-HP, Robert Debre Hospital, 48 Boulevard Serurier, 75019 Paris, France; 2 Neonatology Department, AP-HP, Armand Trousseau Hospital, 26 Avenue du Dr Arnold Netter, 75012 Paris, France; valerie.champion@aphp.fr (V.C.); delphine.mitanchez@aphp.fr (D.M.)

**Keywords:** raw breast milk, cytomegalovirus, milk-acquired infections, preterm infant

## Abstract

Raw breast milk is the optimal nutrition for infants, but it is also the primary cause of acquired cytomegalovirus (CMV) infection. Thus, many countries have chosen to contraindicate to feed raw breast milk preterm infants from CMV-positive mothers before a corrected age of 32 weeks or under a weight of 1500 g. French national recommendations have not been updated since 2005. An audit of the French practices regarding the nutrition with raw breast milk in preterm infants was carried out using a questionnaire sent to all neonatal care units. Diagnosed postnatal milk-acquired CMV infections have been analysed using hospitalisation reports. Seventy-five percent of the neonatal units responded: 24% complied with the French recommendations, 20% contraindicated raw breast milk to all infants before 32 weeks regardless of the mothers’ CMV-status, whereas 25% fed all preterm infants unconditionally with raw breast milk. Thirty-five cases of infants with milk-acquired CMV infections have been reported. The diagnosis was undeniable for five patients. In France, a high heterogeneity marks medical practices concerning the use of raw breast milk and the diagnostic approach for breast milk-acquired CMV infection is often incomplete. In this context, updated national recommendations and monitored CMV infections are urgently needed.

## 1. Introduction 

In the last fifty years, the development of neonatology reversed the prognosis of preterm infants with a weight over 1500 g from a mortality rate of 85% to a survival rate without sequelae of 85% [1,2]. This drop in mortality was accompanied by a steady decrease in severe morbidities [2]. It was mainly related to significant advances in lung maturation, respiratory support, and optimal nutrition [3]. Breast milk is a crucial part in the management of preterm infants with widely documented immunological and nutritional benefits [4]. Its composition adapts to the gestational age at birth to better protect preterm infants and to regulate their immune response [5,6,7,8]. It reduces the risk of infection and inflammatory phenomena, leading to a significant decrease in the incidence of bronchopulmonary dysplasia [9], retinopathy of prematurity [10] and necrotising enterocolitis (NEC) [11]. Nutritional values of breast milk also have a beneficial role in both short and long-term neurological development [12,13,14], and exposure to breast milk antigens promotes the development of tolerance and significantly reduces the risk of allergy and atopic diseases [15,16]. However, the long-term benefits of breast milk on the prevention of leukaemia [17], obesity [18], type 2 diabetes [19], and others are not yet fully assessed. 

The risk of transmitting infections remains a barrier to the use of raw breast milk. To limit this risk, methods have been developed. Freezing reduces the risk of infection (mainly viral risk) without eliminating it, and pasteurisation affects nutritional and antimicrobial properties of breast milk [20]. Whilst there are no differences in neurodevelopmental outcomes in preterm infant fed preterm formula compared with those fed breast milk there is a significantly higher risk of developing NEC with formula [21]. Thus, in light of the improvement of knowledge on the benefits of breast milk, the risk of transmission of the commonly feared infections has been carefully reassessed, and contraindications have been increasingly restricted. With the temporary contraindication of breastfeeding caused by Herpes simplex or Herpes zoster lesions on the breast [22], the only definite contraindication of raw breast milk in developed western countries is maternal HIV-positivity [23,24], and the most discussed one is the maternal cytomegalovirus (CMV)-positivity regarding preterm infants. 

CMV (cytomegalovirus) is reactivated by lactation in the mammary gland with a prevalence greater than 95% and is then transmitted via macrophages, monocytes, and virions present in raw breast milk [25,26]. In the mother milk of full-term infants, CMV is excreted as early as colostrum and during the first three months of lactation. In the mother milk of preterm infants, CMV excretion begins with a lower viral load and the onset of excretion is more variable. It usually begins in the first ten days of life, but may be present from the colostrum [27,28]. The standard diagnostic method of CMV infection was viral isolation on fibroblasts culture from a urine sample, but current polymerase chain reaction (PCR) techniques have better sensitivity and specificity (98.8% and 99.9%, respectively) and can be performed on urine or blood samples [29]. To conclude to a milk-acquired infection, congenital CMV infections should be eliminated by a negative CMV research on a blood or urine sample taken within the 21st days of life or on a salivary sample taken within the 21st days of life and before nutrition by unpasteurised breast milk [29,30]. In the case of CMV infection diagnosed after the 21st day of life, the positivity of the PCR could no longer differentiate congenital and postnatal infections. Then, only a negative CMV PCR on a specimen collected before the 21st day of life (cord blood or dried blood spots collected on blotting paper for newborn screening program) can eliminate the diagnosis of congenital infection. The reactivation of the virus in breast milk can be confirmed the same way, by viral isolation or PCR done on milk sample [31]. In the 1990s to 2000s, studies demonstrated that, in children born before a corrected age of 32 weeks or below a weight of 1500 g, the CMV transmission rate was over 50% in the first three months of life [27]. Moreover, 50% of preterm infants had symptomatic infections, and 15% of these infections were severe [26]. The main symptoms were apnoea, bradycardia, pneumonia, hepatitis, gastrointestinal tract symptoms, and haematological signs (thrombocytopenia, neutropenia, and lymphocytosis). These infections appeared between four and eight weeks of life and were responsible for significant clinical degradations that could be life-threatening, whereas the level of C-reactive protein remained low (10 to 20 mg/L). This clinical situation was called “sepsis-like”. Thus, the international guidelines agreed not to feed preterm infants from CMV-positive mothers with raw breast milk before a corrected age of 32 weeks or below a weight of 1500 g [32,33,34].

In the 2010s, retrospective studies and reviews of the literature reassessed the risk of milk-acquired severe CMV infections and the prognosis of affected infants. The risk associated with symptomatic infections and “sepsis-like” were estimated to be low [35]. In particular, the risk of neurological sequelae (cognitive and motor) was similar to that of preterm infants without a history of postnatal CMV infections [25]. Therefore, since 2012, the American Academic of Pediatrics recommends nutrition with raw breast milk for all preterm infants [36]. However, publications have rapidly reported cases with severe “sepsis-like” and severe enteropathies suggestive of atypical NEC [37,38,39,40]. Several fatal cases have been reported [41,42]. Moreover, since 2015, large cohorts have shown that the incidence of bronchopulmonary dysplasia was significantly higher in infants with postnatal CMV infection [43]. The absence of long-term consequences has also been questioned [28].

Since 2005, the French recommendations maintained to not feed preterm infants from CMV-positive mothers with raw breast milk before a corrected age of 32 weeks or below a weight of 1500 g. The breast milk must then be pasteurised before its administration [32]. This recommendation is problematic for several reasons. Routine CMV screening of pregnant women is not recommended in France [44], and not all neonatal units have access to pasteurisation. Furthermore, several cases of postnatal breast milk-acquired CMV infections in infants fed raw breast milk before a corrected age of 32 weeks or below a weight of 1500 g have been published by French neonatal units [42,45,46]. Thus, this study aimed to evaluate the French national current clinical practices about breast milk nutrition of preterm infants, to carry out a first national census of raw breast milk-acquired CMV infections and to check the validity of this diagnosis.

## 2. Methods

### 2.1. Study Design

An observational, transverse, prospective, multicentre, descriptive study was conducted via a questionnaire sent by e-mail to all NICU (neonatal intensive care unit) and neonatal non-ICU, in mainland France and French overseas territories. 

### 2.2. Outcome Measures 

The questionnaire was sent by email to at least one doctor or breastfeeding counsellor from each unit from June 2015 to June 2016. Reminders were sent out every four months for one year as long as there was no answer. The answers were collected by e-mail and by post. The questionnaire was written in French and translated into English for this publication (Figure S1). 

The questionnaire consisted of four parts. In the first part, general data on the neonatal unit were collected. In the second part, the current clinical practices of each service were requested. The information was related to the use of breast milk (frozen, pasteurised, raw mother milk, or donation of breast milk), the promotion of breastfeeding, and the access to a human milk bank. The third part concerned the conditions of use of raw breast milk (maternal CMV status, infants’ term or weight) and the barriers to its use (mainly infectious risks). Finally, the last part of the questionnaire identified diagnosed cases of postnatal CMV infections imputed to raw breast milk (clinical signs and course).

Subsequently, the neonatal units reporting cases of breast milk-acquired CMV infections were contacted again between June 2016 and June 2017 to obtain the hospitalisation reports of the concerned infants.

### 2.3. Data Management 

CMV infections have been classified as “proven”, “highly probable”, “probable”, or “unlikely” breast milk-acquired infections.

CMV infections have been classified as “proven” if the infection occurred in infants from CMV-positive mothers fed raw breast milk with documented CMV reactivation in breast milk and without any other mode of transmission possible. A congenital infection must have been rejected. Both viral isolation culture and PCR methods were accepted to reject the congenital infection and confirm the infection in the blood or the urine of the preterm infant and to confirm the CMV reactivation in breast milk. Other possible modes of transmission had to be eliminated (PCR on residual blood from transfusions), and no other cause of infection must be found.

CMV infections have been classified as “highly probable” if the infection occurred in infants from CMV-positive mothers fed raw breast milk, but one of the following items was not documented: reactivation of CMV in breast milk, elimination of all other possible modes of transmission, and evidence of absence of congenital infection except mothers’ CMV-positivity prior to pregnancy.

CMV infections were classified as “probable” if the infection occurred in infants from CMV-positive mothers fed raw breast milk, but two of the following items were undocumented: reactivation of CMV in breast milk, elimination of all other possible modes of transmission, and evidence of absence of congenital infection except mothers’ CMV-positivity prior to pregnancy.

CMV infections have been classified as “unlikely” for other cases.

### 2.4. Statistical Analysis

The results for the quantitative variables were expressed in median (minimum—maximum). The results for the qualitative variables were expressed in numbers and percentages.

## 3. Results 

**Figure 1:**
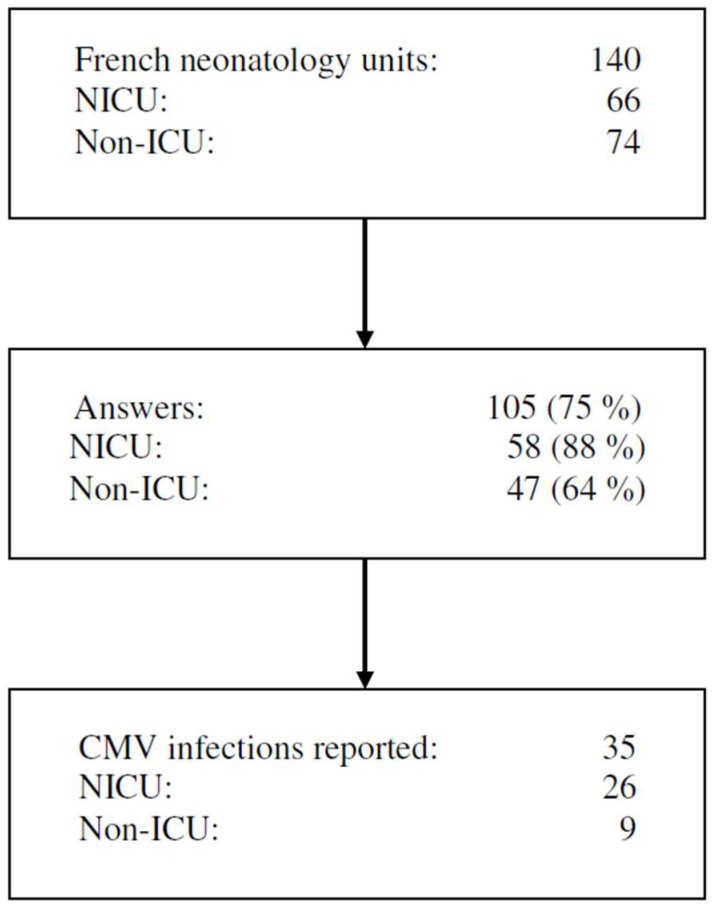
Flowchart. NICU: neonatal intensive care unit; ICU: intensive care unit; CMV: cytomegalovirus.

The questionnaire was completed by 105 neonatal units including 58 NICU (88%) and 47 non-ICU (64%), representing an overall response rate of 75% (Figure 1). The participation rate was evenly distributed across regions, ranging from the smallest to the largest unit (Table S1).

### 3.1. Current Clinical Practices

Ninety percent of NICU and 67% of non-ICU reported promoting breast milk nutrition with 70% of NICU and 63% of non-ICU having a breastfeeding counsellor, but only half of them with protocols to help initial breastfeeding. The storage methods of breast milk were freezing in 17 units, pasteurisation in 32 units, and both methods in 47 units.

The 36 neonatal units located in hospitals with a human milk bank responded to the questionnaire. Fifty-six other neonatal units (53%), including 35 NICU, had access to a human milk bank outside their hospital. Thirteen services (12%) reported not having access to a human milk bank. Among them, six neonatal units (5 NICU) were in overseas territories. The two NICU of the Reunion (overseas territory) were the only units to report the use of freeze-dried women’s milk from the French human milk bank of Marmande. 

Among the 92 neonatal units that had access to a human milk bank, if the infant’s mother milk was unavailable, 87 (95%) routinely used pasteurised women’s milk instead of formulas for preterm infants before a corrected age of 32 weeks. This corresponded to 51 NICU and 36 non-ICU. All NICU systematically used pasteurised women’s milk before a corrected age of 30 weeks, except for a unit that used it systematically only for infants before 28 weeks. For infant over a corrected age of 32 weeks, 37 units (35%), including 19 NICU, used pasteurised women’s milk for initial trophic nutrition, nutrition for infants with significant in utero growth retardation (birth weight <10th percentile and less than 1500 g) or for children with congenital digestive disorders, current digestive disorders, or renal failure.

### 3.2. Raw Breast Milk Use

**Figure 2:**
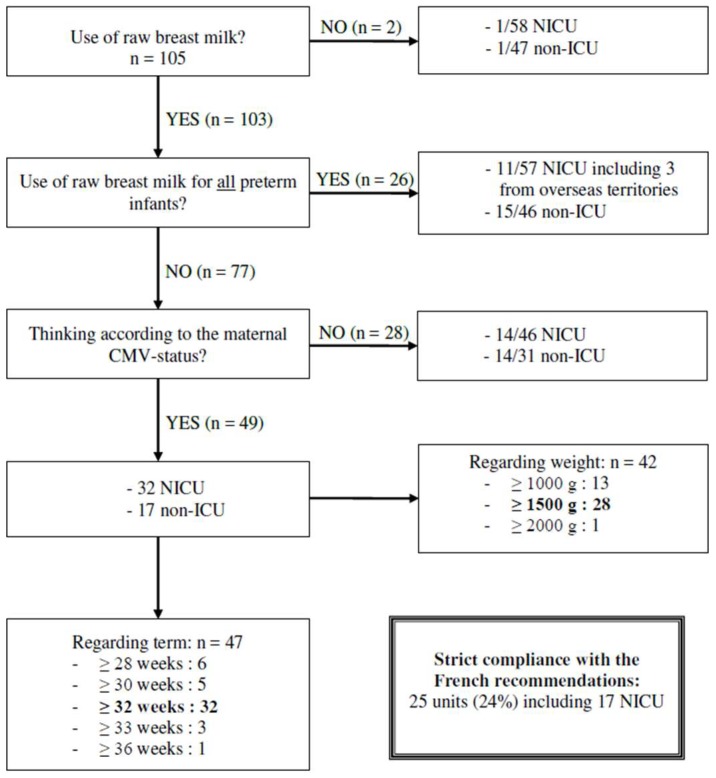
Summary of the current use of raw breast milk in France.

Only two among 105 units declared never to use raw breast milk. Among the 103 units that used raw breast milk, 25% neonatal units fed all infants with raw milk, regardless of term, weight and the mother CMV status (Figure 2). Among them, seven units did not have access to a Human milk bank (including three overseas territories), and eight NICU decided to feed all preterm infants with raw breast milk, despite access to a Human milk bank.

Seventy-three percent of the units used raw breast milk based on the infants’ term or weight. Among them, 49 units gave raw milk according to the CMV maternal status. Thus, all infants born from CMV-negative mothers received raw breast milk from birth. On the other hand, in the case of maternal CMV-positivity, 32 units gave breast milk from a corrected age of 32 weeks, but some gave it either later or sooner (Figure 2). Although 1500 g was the most common weight limit used, 1000 g was also widely used, associated with a term limit of 28 weeks. Twenty-eight units gave raw breast milk based on infants’ term, but not according to the maternal CMV status. Consequently, even infants born from CMV-negative mothers did not receive raw breast milk before a defined term or weight. The clear majority (21 including 11 NICU) gave raw breast milk from 32 weeks to all preterm infants. Other units gave it from terms ranging from 28 weeks to 35 weeks. Overall, 25 units (24%) complied with the French recommendations.

Among the 77 units that did not give unconditionally raw breast milk, 95% reported that the risk of transmission of infectious diseases was the main barrier. The most feared infections were caused by CMV (56 units including all NICU), HIV (43 units including 26 NICU), bacterial infections led by those caused by *Staphylococcus aureus* (33 units including 20 NICU) and HTLV-1 (10 NICU). Five units also reported that the current French recommendations were the principal barrier to the use of raw breast milk. Units that did not give raw breast milk before a corrected age of 32 weeks and below 1500 g, regardless of the maternal CMV-status, highlighted the contradiction of the French recommendations as the maternal CMV serology is not recommended during pregnancy. On the other hand, those who gave raw breast milk to all infants, regardless of their term, weight, and their mother’s CMV-status, justified this approach by the numerous studies on the benefits of breast milk and the latest American recommendations. Other units pointed out that the absence of a human milk bank was of great importance in their decision and that it could have been otherwise. Moreover, some units changed their practices because of cases of severe postnatal infections. 

### 3.3. Reported Postnatal CMV Infection Attributed to Raw Breast Milk Nutrition

Twenty-one units (20%) (17 NICU and 4 non-ICU) reported a total of 35 cases of postnatal CMV infections thought to be transmitted via raw breast milk between 2013 and 2016. Eight infants (23%) had asymptomatic infections, 11 (31%) had moderate signs (hepatic cytolysis, thrombocytopenia), and 16 (46%) had significant signs including 10 infants (29%) with “sepsis-like” infections. Two infants died in NICU during the infection, and another infant died a few months later from complications of this infection. Seventeen hospitalisation reports were obtained, including the reports of two of the three deceased infants and were classified as “proven”, “highly probable”, “probable”, and “unlikely” (Table 1).

The five cases classified as “proven” infections were fed raw breast milk before their second week of life. The congenital origin of the infection was eliminated by a CMV PCR negative on the dried blood spots of the newborn screening program for all of them, excepted one infant with an intrauterine growth restriction who had negative research of CMV done on urine sample during his first week of life. The mean gestational age of birth of these children was 27 weeks (26–29 weeks). Two children had an asymptomatic infection diagnosed for one because of the symptomatic infection of his twin, and for the other during a pilot study. One infant received blood transfusions because of a win-to-twin transfusion syndrome with negative CMV PCR done on the residual blood (patient 1). His autopsy found typical CMV lesions in all organs including the entire digestive tract. Another infant who suffered from “sepsis-like” and NEC showed a CMV PCR positive on peritoneal liquid. 

The seven cases classified as “highly probable” were all fed raw breast milk before their second week of life. When it was done, the congenital origin of the infection was eliminated by a CMV PCR negative on the dried blood spots of the newborn screening program. The children had a gestational age of birth between 25 and 33 weeks. Half of the infections were discovered on biological abnormalities. The mother of the twins born at 33 weeks with a weight over 2000 g was suffering from a CMV mastitis. Only two patients received treatment by ganciclovir: one died (patient 8), and the other showed numerous CMV reactivation (patient 10). The histological examination of the ileocaecal resection of patient 8 showed intense necrotic and pan-parietal inflammatory lesions with typical CMV lesions. This infant presented a persistent hepatocellular insufficiency associated with significant thrombocytopenia requiring numerous platelet transfusions. Four months later, during surgery for restoring the continuity of the gastrointestinal tract, his clinical condition deteriorated rapidly, and he died in the following hours. 

The three cases classified as “probable” were all fed through raw breast milk from their first week of life. In these cases, even if the mothers were CMV-positive before pregnancy, the congenital origin was not eliminated, and the reactivation in the mother milk was not proven. The diagnostic was done after two months of life in the two cases.

Two reported cases considered as milk-acquired CMV infections were classified as “unlikely”. Based on their history, they were probably congenital infections. One did not receive his mother milk but women pasteurised milk. For the second one, the CMV PCR done on the mother milk was negative.

## 4. Discussion 

As a result of a high rate of participation, this work offers a global vision of clinical practices in France. All neonatal units recognised the fundamental issue of promoting breastfeeding and emphasised the importance of individual and adapted care, as shown by the higher importance given to the breastfeeding counsellor compared with the establishment of breastfeeding protocols. Regarding raw breast milk, 24% of units strictly complied with the French recommendations and 20% applied the same limits to all preterm infants, regardless of maternal CMV-status, whereas 25% of neonatal units fed all preterm infants unconditionally with raw breast milk. Most neonatal units believed that the French recommendations are outdated. Their current protocols were the result of reflections including French recommendations [32], recommendations from authorities of other countries [36] or by French experts [47], recent literature, possible access to a human milk bank, and their clinical experience. It resulted in a variety of protocols ranging from raw breast milk nutrition for all preterm infants to non-use of it before a corrected age of 36 weeks or below a weight of 2000 g.

These protocols were mainly based on the fear of severe breast milk-acquired CMV infections in preterm infants. However, this audit shows that the diagnostic approach to conclude such an infection was often incomplete. Out of the 17 hospitalisation reports obtained, the diagnosis was confirmed in only five cases. In the group of infections classified as “highly probable”, the missing step was most often the elimination of congenital infection. In the case of infections classified as “probable”, the two missing elements were both the elimination of congenital infection and the confirmation of the CMV reactivation in breast milk. The diagnosis was always made in the first months of life when the information can be retreived. Indeed, the retrospective way to eliminate a congenital infection is to perform a CMV PCR on a sample taken before the 21st day of life. In France, blotting papers for the newborn screening program are kept for 18 months. Viral DNA testing on the dried blood spots collected on this blotting paper could be done up to five years after birth [48] and the technique to perform a CMV PCR on it has evolved to improve the sensitivity and specificity to 99.9% [49,50]. Thus, the congenital origin of a CMV infection can be confirmed or denied. The second missing element was the proof of CMV reactivation in the mother milk. CMV excretion occurs from the first to the eighth week with a peak of viral load between the third and the fifth week. Freezing decreases the viral load while preserving the viral DNA. Thus, this research can be conducted afterwards, including that on frozen milk. 

**Figure 3:**
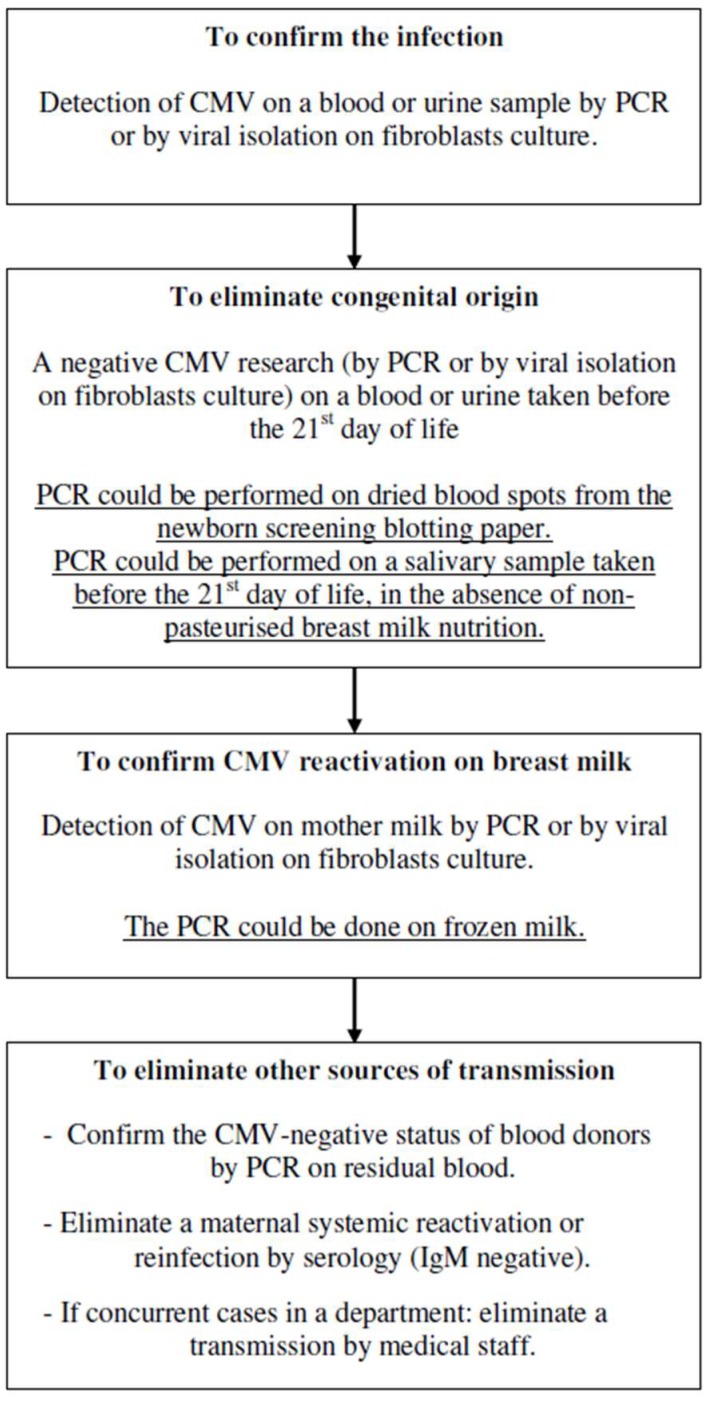
Possible exhaustive diagnostic approach of milk-acquired CMV infections. PCR: polymerase chain reaction.

In the absence of an exhaustive diagnostic approach, it is impossible to know the exact number of postnatal breast milk-acquired CMV infections, as well as their risk factors and prognosis. A possible exhaustive diagnostic approach is presented in Figure 3. The only mode of transmission that is not eliminated by this approach is perinatal transmission when passing the birth canal. Few studies have investigated this mode of transmission, but they showed a near-zero risk in term infant as in preterm infants [26]. However, this approach investigates and eliminates all other sources of transmission. In the 2000s, prospective studies focused on eliminating congenital origin and confirming postnatal infection, without systematically eliminating the risk of transmission through blood products or through confirming the CMV reactivation in breast milk. This fact has been emphasised in the review of the literature by Kurath et al. in 2010 [25] and a meta-analysis conducted in 2017 by Lanzieri et al. [35]. The latter analysed all the studies carried out since 1980 in English, French, Spanish, and Portuguese. Its inclusion factors were known old maternal immunity, birth before a gestational age of 32 weeks or below a weight of 1500 g, elimination of congenital infection, confirmation of postnatal infection, and accuracy of the preservation mode of the breast milk received (pasteurised, frozen, or raw). Only 17 studies conducted between 2001 and 2011 could be included, and elimination of transmission via blood products was analysed, but was not an inclusion factor as few studies explicitly excluded it. These studies underlined the difficulty of analysing the results, considering the heterogeneity of the international recommendations and practices. 

In our study, infants affected by CMV infections were born between 25 and 33 weeks and fed raw breast milk before their second week of life. Infections occurred between the 15th and 70th day of life. All reported cases of severe infections (“sepsis-like”, ECUN, deaths) involved infants born before 30 weeks of age, except for one twin born at 33 weeks with a mother suffering from CMV mastitis. Eight cases involved infants born before 28 weeks with infections occurring until a corrected age of 36 weeks. Studies and reviews of the literature seem to agree that children born before a corrected age of 28 weeks or a weight below 1000 g are at higher risk of developing severe infections, and that 80% of “sepsis-like” concern infants born before 26 weeks [27,41]. One of the reasons is probably the reduced transplacental passage of protective maternal antibodies before the end of the 28th week [51]. The risk of severe infection appears to be increased if preterm infants receive raw breast milk during the first month of life and have comorbidities [52,53,54]. A group of French experts worked on the use of raw breast milk to harmonise practices and wrote the “First Recommendations for the Use of Raw Milk” [47]. The experts provided advice on all crucial steps in breastfeeding, from breastfeeding promotion to protein fortification and viral and bacteriological infectious contraindications. Regarding CMV, the contraindication only concerns infants born from CMV-positive mothers before a corrected age of 28 weeks or below a weight of 1000 g. For these infants, although raw colostrum can be administered within the first 2–3 days of life, milk must be pasteurised up to a corrected age of 31 weeks and 6 days to protect children for at least the first month of life. Six NICU followed these recommendations. However, this expert opinion, like the current French recommendations, raises the problem of CMV screening in pregnant women. The main argument for not recommending this non-targeted screening is the lack of effective treatment for congenital infections. However, this screening would promote hygiene measures to CMV-negative women [55] and facilitate compliance with the French recommendations concerning the nutrition of preterm infants with raw breast milk. Moreover, although no curative treatment exists, early treatment of infants born with congenital infection can reduce the rate of neurosensory sequelae [56]. 

Another issue raised by this study is the link between milk-acquired CMV infection and NEC. In our study, more than half of severe postnatal CMV infections were associated with NEC. The autopsy finding of one infant showed specific CMV lesions in the digestive tract [42], as the histological examination of the ileocaecal resection of a second infant, and a CMV PCR was positive on the peritoneal liquid of another infant. NEC is an acute inflammatory reaction with necrosis of the digestive tract and is the leading gastrointestinal cause of morbidity and mortality in preterm infants with, in very low birth weight, an incidence estimated at 11% [57]. Surgery is required for 50% of them and 35% die [57]. In CMV infection, the involvement of the digestive tract is mainly described in immunocompromised patients where the entire digestive tract can be affected and can lead to digestive perforation with a poor prognosis [58]. Studies have shown that raw breast milk nutrition significantly reduces the risk of NEC [58]. However, the link between NEC and postnatal CMV infection transmitted via breast milk (and thus via the digestive tract) is controversial. Although many case reports highlight this association [37,42], some prospective studies have shown no link between NEC and viral infections [59], while others have shown a significant incidence of CMV infections in acute digestive tract pathologies in preterm infants [57]. The digestive manifestations associated with a postnatal or congenital CMV infection appear to be diverse and include, in term or premature infants, NEC (including atypical) or digestive perforations as volvulus in low birth weight infants [60].

Our study has a major limitation. The questionnaire was essentially empirical and could be completed by a physician, a nurse, or a breastfeeding counsellor. The high level of participation and its even distribution across regions should provide an overview of French practices and enable an optimal census of diagnosed milk-acquired CMV infections. However, we faced a reporting bias and probably an underestimation of the cases. While many units have reported milk-acquired CMV infections, half have finally agreed to send us the hospitalisation reports. Moreover, some known cases have not been reported. The human milk bank of Ile-de-France is often consulted to determine the probability that CMV infections are milk-acquired infections. In 2015–2016, it confirmed four cases. These cases concern four neonatal units who responded to the audit, but none of them has reported the infections in our questionnaire. These cases, like most of the cases reported without an obtained hospitalisation report, occurred in neonatal units using raw breast milk before a corrected age of 32 weeks or below a weight of 1500 g. On the other hand, units that have turned back their practices because of the occurrence of milk-acquired CMV infections, or that continue voluntarily to not follow the French recommendations, have sent us the hospitalisation reports.

## 5. Conclusions

In the absence of an efficient technique to eliminate infectious risk while preserving the nutritional and immunological values of breast milk, consensus on the use of raw breast milk in preterm infants is needed. The French recommendations are indeed too restrictive but, given the heterogeneity of clinical practices and the likely underestimation of infectious risk, new recommendations seem challenging to formulate. The creation of a national registry of milk-acquired CMV infections with a structured diagnostic approach could be an effective way to assess the real infectious risk; identify a population at risk; and, in few years, write national recommendations. These recommendations will have, among others, to rule on the knowledge of maternal CMV status during pregnancy or in preterm births, as well as on the best screening methods for infants. 

## Figures and Tables

**Table 1:** Infection cases.

**“Proven” Infections**	**Term at Birth**	**Weight at Birth**	**Age at Diagnosis (Day)**	**Symptoms**	**Missing Information**	**Source of Reference**
1	27 weeks 4 days	550 g	50	“Sepsis-like”, NEC, death	/	Lopes et al., 2016
2	27 weeks 4 days	1000 g	50	Asymptomatic	/	Lopes et al., 2016
3	26 weeks	810 g	70	“Sepsis-like”, NEC,	/	This study
4	27 weeks	900 g	60	“Sepsis-like”, NEC	/	This study
5	29 weeks	1200 g	53	Asymptomatic	/	Croly-Labourlette et al., 2006
**“Highly probable” infections**						
6	25 weeks 5 days	900 g	36	Thrombocytopenia, hyperleukocytosis	CMV PCR on residual blood from transfusions	This study
7	27 weeks	/	30	“Sepsis-like”	Elimination of congenital origin	This study
8	27 weeks 5 days	950 g	41	“Sepsis-lik”, thrombocytopenia NEC, death	Elimination of congenital origin	This study
9	28 weeks	1125 g	60	Thrombocytopenia	CMV reactivation in breast milk (stopped before)	Boumahni et al., 2014
10	30 weeks	1500 g	15 and 40	Cholestasis “Sepsis-like”	CMV reactivation in breast milk	Radi et al., 2007
11	33 weeks	>2000 g	20	“Sepsis-like”, NEC	Elimination of congenital origin	This study
12	33 weeks	>2000 g	20	Adenopathies	Elimination of congenital origin	This study
**“Probable” infections**						
13	25 weeks	570 g	90	Unconfirmed hearing loss	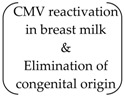	This study
14	32 weeks	>2000	35	“Sepsis-like”		This study
15	32 weeks	1950	60	Severe leukopenia		This study

CMV: cytomegalovirus; PCR: polymerase chain reaction; NEC: necrotising enterocolitis.

## Supplementary Materials

The following are available online at http://www.mdpi.com/2072-6643/10/8/1119/s1, Figure S1: Translation of the questionnaire in English, Table S1: Demographic data.
