# Supplementary material for: Nutrition of Preterm Infants and Raw Breast Milk-Acquired Cytomegalovirus Infection: French National Audit of Clinical Practices and Diagnostic Approach
Source: Nutrients. 2018 Aug 18;10(8):1119. doi: 10.3390/nu10081119 (PMC6115774; doi:10.3390/nu10081119)
Supplement: Supplementary file 1 [file nutrients-10-01119-s001.pdf]

Supplementary Materials: Nutrition of Preterm Infants and Raw Breast Milk-Acquired Cytomegalovirus Infection: French National Audit of Clinical Practices and Diagnostic Approach

Anne-Aurelie LOPES [1,*], Valerie CHAMPION [2] and Delphine MITANCHEZ [2]

AUDIT on the nutrition of preterm infants with raw breast milk and the milk-acquired CMV infections.

In neonatology, breastfeeding is a crucial part in the management of preterm infants. International recommendations agreed on the contraindication to feed with raw breast milk preterm infants from CMV-positive mothers before a corrected age of 32 weeks or under a weight of 1500 g. However, since 2012, the American Academic of Pediatrics recommends nutrition with raw breast milk for all preterm infants, considering that the low risk of severe milk-acquired CMV infection does not offset the benefits of raw breast milk.
Although the French recommendations maintain the contraindication, what are the current practices in France?

General data:

Neonatal unit: ❑ NICU ❑ non-ICU
Hospital:
Approximate annual hospitalisations:
≤ 32 weeks:
≤ 1500 g:

Current practices:

Use of frozen milk: ❑ Yes ❑ No
Use of pasteurised milk: ❑ Yes ❑ No
Use of raw milk: ❑ Yes ❑ No

Promotion of breastfeeding in the unit? ❑ Yes ❑ No
Presence of a breastfeeding counsellor? ❑ Yes ❑ No
Established protocol to help initial breastfeeding? ❑ Yes ❑ No

Access to a Human milk bank? ❑ Yes ❑ No

If the infant's mother milk is unavailable, is Human milk systematically used for infants before a corrected age of:
- <30 weeks? ❑ Yes ❑ No
- <32 weeks? ❑ Yes ❑ No
- <33 weeks? ❑ Yes ❑ No
- <34 weeks? ❑ Yes ❑ No

Comments:

Use of raw breast milk:
Use of raw breast milk (when it is used):

Unconditionally on all preterm infants: ❑ Yes ❑ No
According to the CMV maternal status? ❑ Yes ❑ No
Based on the infants' term: ❑ Yes ❑ No
If yes: which term? ❑ >30 weeks ❑ >32 weeks ❑ >33 weeks ❑ >34 weeks
Based on the infants' weight: ❑ Yes ❑ No
If yes: which weight? ❑ >1000 g ❑ >1500 g ❑ >2000 g

Comments:

What are the obstacles to the use of **raw** breast milk?
Risk of transmitting infections? ❑ Yes ❑ No
Which ones? ❑ CMV ❑ HIV ❑ Others: …….
Others: ……………………………………………………………………………

Comments:

Postnatal CMV infections
Did milk-acquired CMV infections have been diagnosed in the unit?
❑ Yes ❑ No
If yes:
The infection was: ❑ asymptomatic ❑ with mild signs ❑ with severe signs ❑ "sepsis-like" symptoms

The evolution was: ❑ good ❑ bacterial coinfection ❑ sepsis ❑ death

Comments:

Thanks for participating!

Figure S1. Translation of the questionnaire in English.

Table S1. Demographic data.

	NICU (*n* = 58)	Non-ICU (*n* = 47)
Overall hospitalisations	650 (140–2700)	400 (150–1000)
Hospitalisations of infants born before a corrected age under 32 weeks	125 (20–400)	30 (0–250)
Hospitalisations of infants born below a weight of 1500 g	115 (16–400)	26 (0–250)

The results are represented by the median and the extremes (minimum—maximum).
